# The Following Cases of Small-Pox Subsequent to Vaccination, Occurred in the Family of Mr. Brown, Surgeon, Blackfriars' Road, and Were Drawn up and Sent for Publication by Himself

**Published:** 1813-04

**Authors:** 


*For the Medical and Physical Journal.*

*The following* CASES *of* SMALL-POX *subsequent to* VACCINATION, *occurred in the* FAMILY *of* MR. BROWN, SURGEON, BLACKFRIARS' ROAD, *and were drawn up and sent for* PUBLICATION *by* HIMSELF.

FRANCES BROWN, seven years of age, the subject of the following case, was vaccinated by Dr. Walker, when six weeks old, at the central house of the Jennerian Society, in Salisbury-square. The insertion, I conceive, went through its stages correctly, or I should not have remained satisfied of her future security against the variolous infection. I am not aware, at this distance of time, that any were inoculated from her, certainly not more than one or two, and then by taking the lymph in a way least likely to disturb the progress of the pock. It is, with me, an invariable rule to leave one vesicle entire.

Mrs. Baker, at whose school she was, describes her to have been in the most perfect health and spirits on Saturday, November 14th. On Sunday (15th), she appeared heavy, and not disposed to take her food. Mrs. B. observes, as she had had no stool on that day, she gave her small doses of senna on Monday (16th), two or three times. Though she appeared to vomit that, with whatever else she took during the day, she had two motions, and went to bed much better, and in good spirits. Eleven at night, restless, with more fever than Mrs. B. could ascribe to chicken-pock, a complaint which some of the young ladies in the school had had at that time. Mr. Parrot, in his letter to me on Tuesday morning, describes her as having passed a restless night, with delirium, pulse 140, with the appearance of small-pox. Her bowels, in the course of the day, were copiously evacuated twice, by a powder consisting of three grains of calomel, &c.

On my arrival at Mitcham, four o'clock, Tuesday (17th), I found the child with a temperate skin. Petechiæ about the abdomen, particularly on the lower part, extending to the upper and inner part of the thighs. On the arm-pits, and and some way down the under part of the arms, appeared a continued effusion of bright blood, not like the distinct appearance upon the abdomen and thighs. She passed a copious and purging stool the same evening—had a restless night, with delirium.

Wednesday morning (18th). Petechiæ more diffused, losing their bright red color, and becoming more dull; considerable tenderness about the navel; and vomiting all she took during the greater part of the day. I had ordered a draught consisting of ℥j. of Inf. Rosæ, with five drops of Muriatic Acid, to be taken every four hours, which was approved by Dr. Adams, who saw her at noon. This medicine was continued during the disease, and occasionally taken every two hours when awake. She was ordered, by Dr. Adams, a mixture of ℥j. of Manna and T. Jalap.*, to be given till the bowels should be opened. The first two or three doses were vomited up again. A purging injection, administered in the evening, procured a satisfactory evacuation. Pulse 140. Skin hot and dry, with occasional muttering delirium. She took freely of oranges, lemonade, and other cooling acid diluents. Three drops of laudanum were given at ten at night, without abating the restlessness. The same was repeated at one, and she passed the remaining part of the night tolerably quiet.

In the morning, Thursday (19th), seemed cheerful, and amused with her playthings. Skin more temperate. Eruption more distinct, particularly on the face. The petechial appearance had assumed a brighter color, and was somewhat diminished. Three grains of calomel were given, and the opening mixture repeated. An injection was administered in the evening, which procured a purging stool. Though she awoke in the morning apparently better, the day passed with occasional violent delirium, and once she suddenly jumped out of bed; at other times a muttering, without any intermission of insensibility. Nine in the evening, pulse 130. Four drops of laudanum were given at twelve o'clock without procuring any rest; frequent startings and raising herself up in bed, with constant delirium. At five, four drops more were given, after which she fell almost immediately a-sleep, and remained so at half past eight, when I left her.

Friday (19th). The delirium in the early part of last night was with apparent strength, afterwards with muttering, sometimes intelligible, at others not. Eleven o'clock, Dr. Adams

---

* The imperfect state of the MS. compels us to leave the above passage also imperfect.—EDITORS.

found found her pulse 120; skin temperate; the eruption coming on more slowly and favorably in the face. Ten at night—has passed a composed day, and slept quietly the greater part of it, with very little delirium; with a calm and uninterrupted respiration; was always sensible when awoke to take medicine or nourishment. Has taken, during the day, ℥ifs. Manna, with ʒi. of T. Jalap. in ℥vifs. Aq., and only vomited the last dose. Nine in the evening, had a copious and purgative motion. Ptyalism has been, and continues, copious. The swelling of the face and hands in progression; the eruptions becoming more distinct and prominent, with florid interstices. Pulse 120. Skin temperate.

Saturday morning (21st), eight o'clock. Has slept almost the whole of the night without an opiate. Pulse 120. No delirium. Dr. Adams, about noon, found the appearance of the eruption in the face very favorable; the petechiæ in the abdomen absorbed, and a slight desquamation. Much irritation (apparently the beginning of secondary fever), which continued the greater part of the day, without delirium. Has taken ℥ifs. of Manna, and ʒj. T. Jal. in ℥iv. of water, with the addition of three grains of calomel; which, with the assistance of an injection at nine in the evening, produced a copious and purgative stool. Has slept quietly since; breathes freely. The face properly swelled, the pocks upon it appearing more distinct, and looking like an early maturation of them. Swallows without inconvenience, which she has done through the disease. Eleven at night—pustules upon parts of the face looking brown, and evidently turning.

Sunday morning, eight o'clock, (22d). Has had a good night without an opiate. The face very much swelled, and the pustules evidently turning. Noon—the favorable symptoms continuing. Ten at night—has taken light nourishment during the day with satisfaction. Perfectly sensible, and capable of opening her eyes, which she could not do yesterday, and requiring to be amused. The secondary fever, and its expected alarming consequences, appear so much subdued, as to excite no further apprehension. The pustules of the smaller kind, upon the face, partially drying, and others looking brown and full; those upon the arms larger, full, and more confluent than on the face. The tongue moist, and its crust clearing at the edges, also breaking in the middle. Has taken the same quantity of medicine to-day as yesterday, which produced a copious purgative, and bilious-looking stool. Swelling of the face subsiding. The usual fœtor of small-pox scarcely perceptible, though the eruption is so considerable.

 From

From a review of the above case, the following reflections naturally arise.—Would the patient have recovered from the disease, had she not been previously vaccinated. I am decidedly of opinion that she would not, since, from the commencement of the eruptive fever, which was accompanied with delirium and other symptoms of excessive action, to the appearance of the eruption, was not more than forty hours, and the eruption had a confluent appearance, attended with petechiæ, and other symptoms, all considered unfavorable by gentlemen well acquainted with the disease.

I think it may be fairly asked, was there ever a case of confluent small-pox, accompanied with the worst of symptoms, which terminated in the mild way this child's has done, and so early as the eighth day. The pustules appeared to dry off, the ulcerative process prevented, and but slight appearance of future mark. In addition to the above, the absence of that peculiar fœtor accompanying the latter stages of the disease, all tend to confirm, that, if the cow-pock did fail, in this instance, to protect the child from variolous infection, it disarmed the disease of its danger; and to previous vaccination I shall ever consider that I am now indebted for the life of my child. The vaccine process was regular in all its stages.

To the questions concerning the regular process of the elder child, it may be sufficient to say, she was vaccinated by Dr. Walker. She was under my care and inspection during the whole cure, that, had I not been satisfied, she would have been re-vaccinated. It may be further remarked, that for nearly seven years she had escaped the variolous infection, though exposed to it without any reserve.

---

Ellen Brown, five years and a half old, appeared, on Thursday, Nov. 26th, to be dull, had little or no appetite, looked languid, and passed a restless night. Friday (27th), an increase of the same symptoms, with another almost sleepless night, and considerable thirst; complained of pain in her head and back, with red and watery looking eyes. Saturday (28th), kept in bed the greater part of the day, with considerable symptoms of fever, particularly towards evening, and when out of bed required to be kept near the fire. The same night a scarlet rash appeared in different parts, particularly upon the arms and legs, interspersed with pimples, fewer upon the face, and less evident than upon the arms and legs, passed a restless night, with some delirium. Sunday (29th), evident diminution of fever. Pustules small, distinct, with considerable efflorescence on the arms and legs, and, in proportion as it subsided, the small-pox eruption became more evident evident and distinct, though numerous, having 150 upon the face. She passed through the disease in a mild manner; the eye-lids swelled, but did not close; the throat became sore; and she went through the disease with its usual symptoms; and the pock turned on the seventh day.

Two younger children, which had also been vaccinated, shewed evident symptoms of indisposition, looking heavy for two or three days. The elder had a pustule or two upon the face, which were considered by some medical gentlemen as bearing the character of small-pox. These three children had been vaccinated by myself, and the disease, in each, had gone through its stages correctly.

